# Legal reflections on the evolving role of general practitioners in China’s primary care: an assessment of regulatory strategies

**DOI:** 10.1017/S1463423618000555

**Published:** 2018-08-16

**Authors:** Ziyu Liu, Martin Buijsen

**Affiliations:** 1 Law & Health Care (R & G), Erasmus School of Health Policy and Management, Erasmus University Rotterdam, Rotterdam, The Netherlands; 2 Law & Health Care (R & G), Erasmus School of Health Policy and Management, c/o Erasmus School of Law, Erasmus University Rotterdam, Rotterdam, The Netherlands

**Keywords:** Chinese healthcare system reform, gatekeeping primary care, GP services, legislation, regulatory strategies

## Abstract

**Aim:**

To assess the regulation of the Chinese healthcare system in assisting a nationwide implementation of general practitioner (GP) services.

**Background:**

Along with the perennial problems of unaffordable and inequitable healthcare, a rapidly ageing population and the increasing burden of non-communicable diseases pose challenges to the Chinese healthcare system. Recognising these challenges and to satisfy people’s demands for more and better healthcare, China has initiated a plan, named ‘Healthy China 2030’, based on the findings from a two-year joint study by the World Health Organization (WHO) and the World Bank Group (WBG) in collaboration with Chinese agencies. The Chinese healthcare plan, officially approved in 2016, is an attempt to use the people-centred, integrated care (PCIC) model recommended by the WHO and WBG to shape the Chinese healthcare system. In accordance with PCIC, China began the implementation of gatekeeping primary care by introducing GP services to local communities.

**Methods:**

A comparative analysis was employed to point out the importance of introducing GP services. A systematic assessment was carried out to evaluate the regulatory sector of the Chinese healthcare system, including a critical review of related legal norms and a theoretical exploration of external impediments (eg, cultural attitudes, government capacity and interest groups).

**Findings:**

Results demonstrate that the current regulatory sector of the Chinese healthcare system needs to be improved in order to assist the nationwide implementation of GP services and to strengthen its gatekeeping role. Major deficiencies include the problematic relationship between legal norms and health policies, the lack of effective and consistent new legislation, the low rate of social acceptance, and lack of support from agencies. To address those challenges, this paper recommends that preliminary efforts be devoted, in part, to two changes in the legal structure: enacting a specific law, and creating an independent regulatory oversight body.

## Introduction

Along with the perennial problems of unaffordable and less equitable healthcare, a rapidly ageing population and the increasing burden of non-communicable diseases pose challenges to the Chinese healthcare system (Anonymous, 2011; Huang, 2011; Tang *et al*., 2013; Xiao *et al*., 2014). Recognising these challenges, China initiated a healthcare reform plan in 2016 named ‘Healthy China 2030’ (The World Bank Group *et al.*, 2016). The plan was developed in response to a joint study by the World Health Organization and the World Bank Group in collaboration with government agencies in China. ‘Healthy China’ is oriented towards satisfying people’s demands for more and better healthcare. It is an attempt to reform Chinese healthcare services by adopting the people-centred, integrated care (PCIC) model (World Health Organization, 2016).

In general, the PCIC model requires health planners to divert attention from treatment to preventive care, and from merely curing diseases to satisfying people’s healthcare needs and expectations (World Health Organization, 2015; Wiley, 2016). The backbone of a successful PCIC model is a strong primary care system (Starfield *et al*., 2005; World Health Organization, 2016). Accordingly, ‘Healthy China’ prioritises the rebuilding of the healthcare delivery system at the level of the local community with an attempt to strengthen its gatekeeping primary care. To ensure the accessibility and quality of gatekeeping primary care, general practitioner (GP) services (or family doctor services) have been introduced to replace the traditional hospital-based delivery system. Major reform efforts are devoted towards providing a GP for every household in China and establishing electronic personal health records for each Chinese citizen before 2020 (National Health and Family Planning Commission, 2015).

Although much of the existing literature is centred on evaluating and promoting the performance of GPs in high-income countries, it is still a new concept for low- and middle-income countries like China. Therefore, many questions need to be answered. What services will the GP provide? Why does the Chinese healthcare system need the GP system? Will the gatekeeping primary care in China be strengthened by introducing GP services? What is the proper role of the GP in the Chinese healthcare system? Will the adoption of the GP system expand access to healthcare? Will the adoption of the GP system reduce medical expenses, or slow the rate of escalation, while increasing the efficiency of the Chinese healthcare system? Will the GP strategy relieve the tension and restore the trust between doctors and patients? What is the most effective and feasible way to adapt the GP system to the Chinese healthcare system? Finally, the most important question: is the Chinese healthcare system, or even China’s entire society, prepared to embrace the GP system?

Our study attempts to respond to these questions. First and foremost, the paper affirms that the core and unique function of the GP is to provide front-line and preventive healthcare. By comparing community-based healthcare services designed earlier with the new concept of GP services, the paper acknowledges the necessity of introducing GP services to establish and strengthen the gatekeeping primary care in China, but cautions that the regulatory sector of the Chinese healthcare system may need to be improved before the nationwide transition to GP services. To support this position, the paper appraises the regulatory sector of the Chinese healthcare system, in terms of not only relevant legal norms, but also external factors. Results show that major deficiencies in the regulatory sector include the problematic relationship between legal norms and health policies, the lack of effective and consistent new legislation, the low rate of social acceptance of GP services, and the lack of support for GPs from government agencies. Recognising these challenges, the paper recommends that future efforts be devoted, in part, to the following two legal changes: enacting a specific law, and creating an independent regulatory oversight body.

## GP services: establishing and strengthening the gatekeeping primary care

Mariner once said that ‘the change from medical terminology to market terminology both reflects and encourages conceptualising healthcare [as] a market commodity’ (1998: 3). Likewise, identifying the GP’s role is clearly of great importance, not only for policy-making, but also for further improvement. The GP (or family doctor) has been recognised as someone skilled in a medical discipline, who is as important as, or complementary to, a doctor practising a medical specialty (Olesen *et al*., 2000). Despite the fact that no consensus has been reached on the definition so far, all interpretations should at least reflect the core and unique function of GP services, which is to practise at the front line of healthcare (Franks *et al*., 1992; Yip and Hsiao, 2009b).

### Primary care in China before the introduction of GP services

The current Chinese healthcare system is a hospital-based delivery one where public hospitals, as ‘one-stop shops’, take care of the majority of healthcare demands. At the local level, primary care is supposed to be guaranteed by community health centres (CHCs) and community health stations (CHSs) in urban China, and by township health centres and village health stations in rural areas (Bhattacharyya *et al*., 2011). These medical institutions should, in principle, serve as the gatekeepers to hospitals and specialised medical care, but in reality they fail to play this role.

This ‘failure’ is partially because patients in China can access walk-in services in public hospitals without a referral letter. Even worse, two factors aggravate the problem. One is the current Chinese health insurance scheme, which has covered over 95% of the Chinese population since 2012 (Meng *et al*., 2015: 78). It consists of multiple schemes: the Urban Employee Basic Health Insurance (1998), the New Rural Cooperative Medical Scheme (2003), the Urban Resident Basic Health Insurance (2007), and the Medical Financial Assistance System (2012). In general, these schemes prioritise the reimbursement of inpatient care over outpatient care (Xiao *et al*., 2014). Furthermore, the reimbursement rates of these schemes differ between them. The priority of reimbursement and the different reimbursement rates drive patients to seek inpatient care provided by public hospitals without considering whether it is necessary or not, which, in turn, results in a comparatively low rate of outpatient care provided by the community-based medical institutions. Although the community-based medical institutions also provide limited inpatient care, their major function still focuses on expanding the accessibility of outpatient care. The other impediment to discouraging hospital use concerns the quality of medical services. Compared with the community-based medical institutions, public hospitals are believed to have advanced facilities and better-educated medical professionals. Naturally patients demand high-quality medical services. The lower level of trust in the quality of medical services provided by medical institutions, such as CHCs, causes patients to hesitate to access community resources as their ‘first contact care’ (Bhattacharyya *et al*., 2011: 179).

The combination of easy access to public hospitals and distrust in the quality of community care results in a tricky dilemma: public hospitals are overwhelmed with patients, while community-based medical institutions receive and treat fewer patients. To tackle the imbalance, public hospitals used to cut the number of beds, rather than cooperate with the community-based medical institutions. Unsurprisingly, cutting beds worsened the situation by causing inaccessibility of medical services, particularly tertiary care, and escalating medical expenses.

The public hospital dilemma was exacerbated by ‘drug mark-ups’ (The World Bank Group *et al.*, 2016), which refers to the system by which ‘hospitals receive kickbacks from drug companies for prescribing their products’ (Yip and Hsiao, 2008: 463). This system gives health professionals financial incentives to over-prescribe drugs with high-profit margins. Even worse, the problem of over-prescription results in some patients’ resistance to certain drugs and increases tensions in the doctor–patient relationship (Yip and Hsiao, 2009a; 2009b; Ling *et al*., 2011).

The traditional hospital-based delivery system needs to be changed, with special attention devoted to improving preventive care. In this regard, gatekeeping primary care is of vital importance and should be addressed as the top concern. Either as a supplement or an alternative to the current system of community-based healthcare services, GP services represent a step forward, and should be introduced in China in line with the PCIC model.

### A brief overview of the GP system

Under current conditions in China, the GP system refers to a small group of medical professionals who provide basic medical care, public health and any contractual health management services (National Health and Family Planning Commission, 2016). Regardless of whether the GP is considered a supplement or an alternative to community-based medical institutions, how will the GP system be characterised?

Table 1 outlines GP services and compares them with community-based healthcare services. As shown in Table 1, GP services should include, but not be limited to, ‘common diseases management; immunisation and primary community health prevention; rehabilitation and family planning’ (Kong and Yang, 2015: 89). Furthermore, the qualified GP should, in principle, be fully educated and trained to provide these services, but the system currently permits practising specialists and medical professionals with a background in Chinese medicine to be GPs, in order to address the shortage (Bhattacharyya *et al*., 2011). Moreover, medical expenses should be covered by health insurance mechanisms, the public health budget and patient co-payments. In addition, the regulatory strategy should adopt incentive schemes to encourage patients to use the GP as the first contact for healthcare, and should rely on the power of the local communities to monitor and assess the performance of these doctors. Leadership should also be clarified and strengthened by smoothing communication and fostering collaboration among different government agencies, with an appropriate division of labour. Last but not least, an information-sharing system and an effective referral network are required to improve the performance of GPs.

**Table 1:** Comparative analysis of the current community-based healthcare system and the general practitioner (GP) services under the people-centred, integrated care (PCIC) model

	Current community-based healthcare system	GP services under the PCIC model
Medical providers	CHCs, CHSs, THCs, VHSs	GP Group
Accountable sector(s)	CHCs, CHSs, THCs, VHSs	GP
Major services	CHC example: Disease prevention and control; Healthcare survey and education; Common diseases management; Rehabilitation and family planning	Common diseases management; immunisation and community health prevention; rehabilitation and family planning; any contractual services of health management
First contact care or not	Flexible choice of patients	Strict
Referral method	Allow patient self-refer to higher-level medical institutions	Encourage a strict referral method: ‘1+1+1’ contract model^a^
Relationship with secondary and tertiary hospitals	Weak communication, Loose collaboration, ambiguous division of labour^b^	More integrated collaboration; a clear division of labour
Reimbursement	Less rate of reimbursement	Health insurance schemes; public health budget; co-pay of patients
Regulation (legal)	Government as provider Doctor–patient relationship is mainly regulated by legal norms in public law area	Government as regulator Doctor–patient relationship should be regulated by legal norms in private law area

CHC=community health centres; CHS=community health stations; THC=township health centres; VHS=village health stations.

a National Health and Family Planning Commission of the P. R. China (2016).

b There are roughly three models of collaboration between hospitals and community-based medical institutions: Loose Collaboration Model, Medical Consortium Model, and Direct Management Model. The above three traits are generated from the Loose Collaboration Model since it is employed the more often than the other two models. See Xu *et al*. (2016).

In 2015 the Chinese government introduced the GP concept to limited areas, such as Shanghai (Jing *et al*., 2015), Chongqing (Chen *et al*., 2015) and Guangdong (Kuang *et al*., 2015), to study the impact. A great deal of positive evidence has been reported, especially enhanced and timely medical services and a growing satisfaction with the medical experience. These promising outcomes accelerate the progress of nationwide implementation. Nevertheless, the selected experimental areas are mainly well-developed cities where people enjoy a higher level of social welfare, including education and medical care, than the average person in China.

Situations will be different, or even more complicated, when GP services are extended to the whole nation. Admittedly, it is necessary to introduce the GP to establish and strengthen the gatekeeping primary care in China, but it might turn out to be a problem if the Chinese healthcare system or even the entire society is unprepared. So it is important for the central government to secure a sound legal environment for GP services before nationwide implementation.

## Prepared or not: assessment of the regulatory sector for GP services

Roberts *et al*. (2004) proposed a ‘five-control knob’ framework to measure the achievement of healthcare system reform goals. The control knobs are financing, payment, organisation, regulation and behaviour. By using the regulation knob, we have appraised the current Chinese healthcare system in terms of whether it is prepared for the national rollout of GP services. The appraisal consists of assessing the internal structure and external factors. The internal structure assessment mainly focuses on analysing relevant legal norms, whereas external factors are explored by answering three questions: (1) what are the general and specific cultural attitudes towards regulation of the Chinese healthcare system; (2) do the relevant government agencies have adequate capacity to assure the enforcement of regulations; (3) does the regulatory sector have enough support from relevant interest groups to promote the GP system?

### Assessing internal structure: current effective laws and regulations

Theoretical studies involving legal norms and GPs mainly concentrate on altering individual behaviours in the healthcare sector, both from the perspectives of controlling the over-prescription of drugs and medical malpractice, and dealing with violence against GPs (Sage *et al*., 1994; Allen, 2000; Hoffmann and Tarzian, 2003; Roberts *et al*., 2004; Hesketh *et al*., 2012; Durand *et al*., 2015; Xing *et al*., 2015).

Specifically, Chinese lawmakers have enacted the Tort Law (2009) to control medical malpractice, the Law on Practising Doctors (Revision, 2009) to deter and punish physician misbehaviours, and Amendment (IX, 2015) to the Criminal Law to punish perpetrators of physical violence against medical professionals. Table 2 outlines provisions contained in a select group of relevant laws and regulations covering the important areas of medical practice. It is easy to see that the majority of current legal norms have been effective since 2009. This is partially because a deep reform of the Chinese healthcare system was initiated in that year. In truth, legal norms have served mostly as tools for achieving certain political goals, rather than as a means of independent oversight to monitor and assess the performance of government agencies. This practice has placed the authority of legal norms at risk. Thus, the proper role of legal norms in improving the Chinese healthcare system needs to be re-affirmed deliberately.

**Table 2:** Effective legal norms concerning important areas of medical practice^a^

	Medical malpractice	Confidentiality	Informed consent	Physical violence
Issuing Authority: Standing Committee of the National People’s Congress^b^				
Law on Practising Doctors (Revision, 2009)	Article 29 Article 37–38 Article 42	Article 37 (9)	Article 26	Article 40
Tort Law (2009)	Article 54–64			
Amendment (VII, 2009) to the Criminal Law		Article 253 (A)		
Amendment (IX, 2015) to the Criminal Law				Amend Article 290
Public Security Administration Punishments Law (Amendment, 2012)	Article 72			Article 23 (1)
Law on Prevention and Treatment of Infectious Diseases (Amendment, 2013)		Article 12 Article 68 (5)		
Issuing Authority: State Council				
Regulation on the Handling of Medical Accidents (2002)		Article 10		
Regulation on the Prevention and Treatment of HIV/AIDS (2006)		Article 39 Article 56		
Issuing Authority: Department Regulatory Documents				
Notice of the Ministry of Health on Issuing the Basic Norms for Electronic Medical Records (for Trial Implementation, 2010)		√		
Regulations on Medical Institutions for Medical Records Management (2013)		√		

a English translation of laws and regulations is from pkulaw.cn http://en.pkulaw.cn/. Accessed 7 April 2017.

b The 2013 National People’s Congress announced the establishment of the National Health and Family Planning agency to replace the former Ministry of Health and National Population and Family Planning Commission.

The fragmented legal norms may also be incompatible with the concept of GP services, since they were issued to deal with the problems generated by the hospital-based delivery system. Put differently, the current version of Chinese healthcare was designed with less attention to support integrated care, and there was hardly any attempt to consolidate the fragmented legal norms. So the fragmentation of legal norms needs to be taken seriously before any substantial steps are taken to implement GP services nationwide.

The current legislative method also has a deficiency. Regulations are more likely to be issued in the domain where bad behaviour causes the worst consequences (Roberts *et al*., 2004). Consequently, certain domains are overwhelmed by overlapping legal norms, while some domains are regulated either implicitly or far from adequately. For instance, informed consent, which is the essential element of patient rights, is merely protected by Article 26 of the Law on Practising Doctors, which stipulates: ‘Doctors shall tell the patients or their family members the patients’ condition truthfully. However, care shall be taken to avoid an adverse impact on the patient. Doctors shall get the approval from the hospital and the consent of the patient or family members before conducting clinical treatment on an experimental basis’ (Article 26, Law on Practicing Doctors, 2009 Revision). Thus, doctors have a ‘notification responsibility’ (Meng *et al*., 2015: 62) to honestly provide medical information (eg, illness conditions, risks and treatment options) to patients and their families, but there is no explicit regulation on the extent and scope of discourse. Since no consensus has been reached regarding whether informed consent is a legal mandate or merely an ethical requirement, this implicit way of regulation seems to be fine for the current Chinese healthcare system (Rao, 2008). Nevertheless, it would no longer be sufficient after transition to the GP system, since the good performance of GPs relies on a higher level of mutual trust and a closer, contractual doctor–patient relationship. In the new context, informed consent should be affirmed as a mandatory duty, and should be regulated strictly in terms of both extent and scope. Therefore, optimisation of the legislative method to cover all related domains needs to be addressed in order to regulate GP services effectively.

In summary, the regulatory sector of the Chinese healthcare system is unprepared in terms of its internal legal structure. Before the nationwide implementation of GP services, relevant government agencies need to make a joint effort to alter the problematic role of legal norms, to consolidate fragmented legal norms, and to optimise the legislative method.

### Exploring external factors: cultural attitudes, government capacity and interest groups

Which external factors are impediments to the regulatory changes required for reform of the Chinese healthcare system? According to the ‘five-control knob’ framework (Roberts *et al*., 2004), there are three external factors influencing reform efforts in the regulatory sector: ‘cultural attitudes, government capacity and political support’. In our analysis, we have replaced the third benchmark with ‘interest groups’. Although Roberts *et al*. (2004: 255) have briefly discussed the organised interest groups and their potential influence on the reform efforts, their major concern is not to identify those interest groups, but to use them as a juncture to address the importance of strengthening political support (ie, political skills, regulatory process and effective implementation) in regulation design. However, the aim of this section is to identify the external factors that actually impede the reform of China’s primary care. Thus, we use ‘interest groups’ instead of ‘political support’ with a conviction that the solid alliance that has been formulated between pharmaceutical companies and major hospitals deserves equal attention.

External factors will be explored by answering questions. What is the cultural attitude, generally and specifically, about using regulations to drive the performance of the Chinese healthcare system? Do the relevant government agencies have adequate capacity to assure the enforcement of regulations? Does the regulatory sector have enough support to ensure the effectiveness of the GP system?

#### Cultural attitudes

Roberts *et al*. (2004) concluded that the majority of Chinese citizens are likely to find ways of avoiding regulations, instead of complying with regulations, since they are more vulnerable and are more likely to suffer from rule violations when compared with people living in countries like Denmark. Besides, *yansu* (antipathy to litigation) solidly underpins traditional Chinese culture, which assigns a low priority to resorting to court to resolve disputes (Meng *et al*., 2015: 63). This general cultural attitude may impede the substantive function of legal norms in advancing the performance of GP services.

With regard to the cultural attitude specific to healthcare, Mathers and Huang (2014: 270) point out that ‘the Chinese people tend to seek high-level care even for minor, self-limiting conditions’. To change this attitude, should legal norms take up the responsibility of educating patients to use healthcare resources more reasonably? What about the personal preferences of GPs regarding where to practise healthcare? Should legal norms be issued to influence GPs to serve rural areas? Some countries, such as India, have forced medical graduates to serve resource-scarce areas for a certain period of time (Bhattacharyya, 2014). As for China, however, medical graduates are encouraged, rather than forced, to serve rural areas by some incentive schemes, including a high salary and more opportunities to receive advanced training. Further investigation is needed as to whether the interference of law is appropriate in these personal affairs. After all, both general and specific cultural attitudes are of great importance for the effectiveness of regulations in advancing the performance of the new GP system. Thus, regulators should be fully aware of these attitudes.

#### Government capacity

According to the analysis of Roberts *et al*. (2004: 253), countries with ‘a high-quality civil service, well-functioning police and court systems and effective tax-reporting’ are more likely to achieve success in constructing an effective regulatory agency for their healthcare systems. Using these criteria as benchmarks, China seems to fall short of capacity.

Table 2 shows relevant legal norms concerning practising doctors launched by different levels of authority in China. The Standing Committee of the National People’s Congress has the highest authority in enacting laws and regulations, followed by the State Council and the National Health and Family Planning Commission. Although China is governed by a single party, these central government agencies have a clear division of labour in line with a strict legal order. By the same token, a clear division of labour does not equally imply effective checks and balances. The deficiency of government capacity becomes aggravated in the implementation process, since there is substantial discretion of local governments (Roberts *et al*., 2004). Regulations issued by the central government are presumed to be subject to uneven reactions at the local level, either because of different socio-economic conditions, or due to the uneven organisational capacity of different regions. Therefore, the substantial discretion of local governments should be taken into account by regulators before issuing any regulation of GP services.

#### Interest groups

The reform efforts will confront resistance from formal and informal organised interest groups for various reasons. In the healthcare sector, pharmaceutical companies and major medical institutions – in particular public hospitals – have formed a solid alliance since pharmaceutical incentives, known as ‘drug mark-ups’ (The World Bank Group *et al.*, 2016), have become a popular source of hospital revenue in the Chinese healthcare system. Although this activity has now been recognised as illegitimate and forbidden (The World Bank Group *et al.*, 2016), the solid alliance is not that easy to destroy. Any reform that may pose a threat to vested interests will certainly face great opposition.

A nationwide shift to GP services is believed to be one of those efforts that will engender stiff opposition, since the intention is to change the traditional hospital-based delivery system fundamentally. Likewise, any new regulation proposed in support of GP services will be influenced by these powerful players in the Chinese healthcare system. Regulators should therefore be clear about their expectations regarding those vital players, including their roles, attitudes and potential for compromise. Only by recognising sources of support and opposition realistically can legal norms be issued effectively, with the full engagement of every relevant player.

In summary, the regulatory sector of the Chinese healthcare system is unprepared to address the influence of external factors, such as cultural attitudes, government capacity and organised interest groups. Cultural attitudes, both general and specific, show a rather low acceptance of regulatory engagement with the GP system. Moreover, the substantial discretion of local governments results in self-interested enforcement of regulations. The uneven regional implementation will impede the effectiveness of regulations in protecting and improving the GP system. In addition, players such as public hospitals and pharmaceutical companies lack incentives to engage in reform. How to secure adequate support from them is of great importance for a well-established GP system.

## Recommendation: enact a specific law and create an independent regulatory oversight body

Recognising that the regulatory sector is unprepared for the challenge of implementing the new healthcare system, we propose two ways to improve internal structure and address external factors, respectively. We recommend that, before the nationwide implementation of GP services, efforts should be devoted to, but not limited to, the following two reforms: enacting a specific law, and creating an independent regulatory oversight body.

### Internal reform: enactment of a specific law

Legal norms should be issued and organised to shield people from the potential adverse effects of the GP system. Apparently the current effective legal norms concerning practising doctors are incompatible with the proposed GP services. A review of the laws and regulations, as listed in Table 2, indicates a great need to eliminate fragmentation to prevent conflict between overlapping legal norms.

In the future, steps can be taken to revise the Law on Practising Doctors by amending and inserting articles in line with the requirements for the administration of GP services. Take licensing and accreditation as simple examples. Medical institutions in China, whether focusing on medical treatment or disease prevention, have an affirmative responsibility to measure the performance of all doctors who have registered in their institutions, not only in terms of reporting malpractice and medical accidents (Article 16), but also in terms of providing training and continued education (Article 35). With regard to the proposed GP services, who should take up those responsibilities? In accordance with the National Health and Family Planning Commission of the P. R. China (2016), the GP is an identifiable person with primary responsibility for his or her medical group (Jing *et al*., 2015). Put differently, the GP has to self-report malpractice and medical accidents, which seems difficult or even impossible to realise. Such regulations should therefore be revised with a thorough consideration of feasibility.

Furthermore, the GP system adopts a ‘1+1+1’ contract model, which means that patients are encouraged to sign the contract with one GP group plus one secondary hospital plus one tertiary hospital. This contractual character of the doctor–patient relationship under the GP system deserves special attention. It implies that people living in China need to be empowered and activated in order to choose the best contract. Once again, regulators have to be cautious when adopting new concepts. If GP services are governed by contractual considerations, then regulations have to deal with the unequal positions of GPs and their patients. In this respect, regulations should be formulated with special attention devoted to creating a supportive legal environment, such as securing patients’ right to information, providing an effective complaint process, and other empowering measures [eg, ‘building health literacy, improving self-management skills, cultivating shared decision-making and creating a supportive environment’ (The World Bank Group *et al.*, 2016: 52)].

Moreover, does the GP have the right to choose patients when the contractual relationship implies an exclusive relationship? According to Article 24 of the Law on Practising Doctors, doctors have an affirmative responsibility to take care of patients under emergency situations. Certainly, any emergency case should be excluded from contractual conditions due to humanitarian values. How about other cases? If the GP is entitled to select patients, is there any difference between commercial insurance companies and the GP? Will the rights of doctors or insurance companies limit the accessibility of healthcare for patients who suffer from severe chronic diseases? Since the law will affect everyone, Chinese lawmakers need to consider this issue carefully.

Last but not least, considering the fact that there exists a wide disparity between urban and rural areas in primary care in China (Shi, 1993; Liu *et al*., 1999; Zhang *et al*., 2016), future legislation should pay special attention to assisting the implementation of GP services in the rural areas of China where people are more likely to suffer from lack of medication and qualified personnel in healthcare. However, this raises many other questions, such as should legislation interfere in the career choices of medical graduates for the sake of dealing with the shortage of healthcare personnel in rural China? Questions like this are crucial for future legislation and thus need to be considered fully during legislative design.

### External reform: creation of an independent regulatory oversight body

In general, Chinese healthcare reform has been managed in a multi-leadership style. The government agencies in charge are the National Health and Family Planning Commission, the Ministry of Finance, the National Development and Reform Commission, and the Ministry of Human Resources and Social Security. Each government agency represents its own political constituency, which results in weak interagency communication and collaboration. Reform plans developed in this multi-leadership style are highly likely to fall short in accountability provisions for each sector, and to contain contradictory arrangements. Although a typical example of multi-sector cooperation exists in China, the Patriotic Health Campaign (Meng *et al*., 2015: 15), this cross-sector coordination mainly focuses on protecting and promoting public health services instead of the entire primary care. Given these drawbacks, together with the substantial discretion of local governments and the physician self-reporting dilemma, the enforcement process performs poorly in practice. Successful implementation of the GP system calls for greater attention to the regulatory body. Creation of an independent regulatory oversight body may be a feasible strategy to pave the way for nationwide implementation.

There are two essential elements of the proposed independent regulatory oversight body. One refers to the involvement of professionals with multi-disciplinary backgrounds. Since healthcare reforms depend on consistent systematic efforts, the proposed body should recruit physicians who are able to provide clinical insight due to their front-line role in the healthcare system, economists who can provide input on sustainability and effectiveness, legal professionals who can draft laws and regulations to prevent misbehaviour and shield people from the adverse effects of reform, ethicists who can provide guidance on humanitarian performance, and professionals from social media who can cultivate cultural attitudes by educating people to be responsible and rational beneficiaries of the reform.

Procedural justice is another concern. Formulating an independent regulatory oversight body should not be regarded as an attempt to replace any effective government agency. On the contrary, it should function independently as a bridge to mitigate the conflicts resulting from the multi-leadership style. In addition, the oversight body should not only serve as a surveillance tool, but should also provide an open process for mediating medical disputes.

## Conclusions

As the cornerstone of the PCIC framework, the evolving GP system has been introduced to establish and strengthen the gatekeeping primary care in China. Compared with the previous system of community-based medical services, GP services are believed to perform better in serving the gatekeeper role. The new GP system is promising, not only in terms of restoring trust between doctors and patients, but also with regard to the enhanced referral mechanism, which will contribute to improvement in communication and collaboration among different levels of medical institutions. However, these promising aspects could also produce unexpected adverse effects if the healthcare system, or even the entire society, is unprepared. In this regard, we have considered the current Chinese healthcare system, with special attention paid to the regulatory sector. Results show that major deficiencies in the regulatory sector include the problematic relationship between legal norms and health policies, the lack of effective and consistent new legislation, the low rate of social acceptance of GP services, and the lack of support for GPs from government agencies. Therefore, neither the internal structure nor the external environment of the regulatory sector is prepared. Recognising that a well-developed regulatory sector is of vital importance for the effective nationwide implementation of the GP system, future efforts should be directed, at least in part, towards enacting a specific law and establishing an independent regulatory oversight body. Only after these requirements are met can GP services be implemented effectively on a nationwide scale.
